# Gut feeling: randomized controlled trials of probiotics for the treatment of clinical depression: Systematic review and meta-analysis

**DOI:** 10.1177/2045125319859963

**Published:** 2019-06-26

**Authors:** Viktoriya Nikolova, Syed Yawar Zaidi, Allan H. Young, Anthony J. Cleare, James M. Stone

**Affiliations:** Centre for Affective Disorders, Department of Psychological Medicine, Institute of Psychiatry, Psychology and Neuroscience, King’s College, Denmark Hill, London, SE5 8AF, UK; GKT School of Medical Education, King’s College London, UK; Institute of Psychiatry, Psychology and Neuroscience, Department of Psychological Medicine, King’s College London, UK; National Institute for Health Research Biomedical Research Centre at South London and Maudsley NHS Foundation Trust and King’s College London, UK; Institute of Psychiatry, Psychology and Neuroscience, Department of Psychological Medicine, King’s College London, UK; National Institute for Health Research Biomedical Research Centre at South London and Maudsley NHS Foundation Trust and King’s College London, UK; Institute of Psychiatry, Psychology and Neuroscience, Department of Neuroimaging, King’s College London, UK

**Keywords:** depression, probiotics, randomized controlled trial, systematic review

## Abstract

**Background:**

Recently the gut microbiota has attracted significant interest in psychiatric research due to the observed bidirectional gut–brain communication. A growing body of evidence from preclinical work has suggested that probiotics may be effective in reducing stress and anxiety and alleviating low mood. It is unclear to what extent these effects are seen in clinical populations. We aimed to identify all published evidence on the efficacy of probiotics as treatment for depression in clinically depressed populations.

**Methods:**

Randomized controlled trials of patients with depression where probiotics were used as supplementary or standalone treatment were considered eligible. A literature search with the terms (probiotic* OR *bacteria* OR *Lactobacillus* OR *Bifidobacterium*) AND depress* was performed in PubMed and Web of Science. Data on study population characteristics, treatment effectiveness, tolerability and risk of bias were extracted from eligible studies. A random effects model was used for meta-analyses.

**Results:**

Only three studies met inclusion criteria (229 individuals randomized), two of which administered probiotics as a supplementary treatment to antidepressants and one as a standalone treatment. Upon removal of the latter study from the meta-analysis due to clinical heterogeneity, there was an overall positive effect of probiotics on depressive symptoms (standardized mean difference = 1.371, 95% confidence interval 0.130–2.613).

**Conclusions:**

There is limited evidence for the efficacy of probiotics in depression at present, although there may be a beneficial effect of probiotics on depressive symptoms when administered in addition to antidepressants. Further studies are required to investigate this and explore potential mechanisms.

## Background

Major depression is a common and complex illness that significantly diminishes quality of life.^1^ It is estimated that 216 million people suffered from major depressive disorder (MDD) in 2015 worldwide,^2^ and rates are predicted to increase due to an increase in incidence rates among younger individuals.^3^ MDD is one of the largest leading causes of health-related disability, and the most costly of all mental health disorders.^4^ Currently, most pharmacological treatments target neurotransmitter activity in the brain and are known to have a delayed onset of effect as well as a range of side-effects. Further, it has been estimated that up to 60% of patients with MDD experience some degree of nonresponse to these treatments.^5^ Therefore, identifying novel treatment approaches is of key importance.

In recent years, a complex bidirectional communication between the gut and the brain has been identified, which has attracted significant interest in psychiatric research. The mechanisms underlying the gut–brain axis are thought to span the gastrointestinal tract, central nervous system (CNS), autonomic nervous system, enteric nervous system, neuroendocrine system and immune system.^6^ A growing body of evidence suggests that psychiatric disorders such as MDD may be related to changes in the gut microbiota. For example, preclinical studies with germ-free mice (with no gut bacteria) have shown that these animals exhibit increased stress response and anxiety-like behaviours and deficits in social behaviour and memory – all of which are prominent features of depressive disorders.^7^ These findings have also been replicated in mice given a mixture of antibiotics or with induced bacterial infection.^8,9^ These effects have been shown to be reversed by restoring commensal microbiota or by treatment with probiotics.^7–9^ Other groups have investigated the effect of rodent models of depression on gut microbiota: Barseghyan and colleagues found significantly increased numbers of *Candida albicans* and *Staphylococcus aureus* and decreased numbers of *Lactobacilli* and *Bifidobacteria* in rats after 2 weeks of chronic variable stress.^10^ Similarly, Naseribafrouei and colleagues investigated the relationship between faecal microbiota and depression in patients with MDD, and discovered that MDD was associated with a specific pattern of overrepresentation of the order of bacteria Bacteroidales and an underrepresentation of the family Lachnospiraceae.^11^ Taken together, these findings suggest that gut bacteria may have a role in causing or maintaining depressive disorders, and that probiotics may be a potential novel treatment approach.

Several studies have investigated the mood-altering properties of probiotic supplements in people without a clinical diagnosis of depression, and have found that these can reduce feelings of depression and anxiety, stress and cognitive reactivity to sad mood.^12–15^ These studies have been reviewed extensively elsewhere.^16,17^ The accumulated evidence from these studies supports the preclinical evidence that probiotics may be beneficial for the treatment of low mood. However, for probiotics to be considered a viable treatment option for MDD or other affective disorders, evidence from clinical trials in well-defined clinical populations is needed. The objective of this review was to identify and synthesize all published data from randomized controlled trials (RCTs) on the efficacy of probiotics as treatment for depression in clinically depressed patients.

## Methods

### Criteria for inclusion of studies in the review

Studies considered eligible for this review were peer-reviewed published RCTs where probiotics were administered as an active intervention for patients with clinical depression, and where the effects of probiotics on depressive symptoms were assessed as the primary outcome. Studies must have either targeted patients who were clinically diagnosed with MDD or used an appropriate measurement of depressive symptoms to determine that they are clinically depressed. Studies in which probiotics were administered for the treatment of another disorder (e.g. irritable bowel syndrome) and where effects on depression were assessed as a secondary outcome were deemed not eligible for inclusion.

### Search strategy

The literature search was performed according to PRISMA guidelines for systematic reviews.^18^ The search terms included [probiotic* OR *bacteria* OR *Lactobacillus* OR *Bifidobacterium*] AND [depress*] and the search was limited to human studies. The initial search was conducted on PubMed and ISI Web of Science databases on 11 May 2018 with no restrictions on date of publication. An additional search on Google Scholar was performed to identify any studies that may have been missed. This was further supplemented by reviewing the bibliographies of relevant articles and recent reviews. All search results were evaluated against search criteria independently by VN and SYZ, with discrepancies resolved by JS. Data extraction of basic study characteristics, measures of treatment effect and tolerability, and quality assessment was conducted by VN and SYZ and reviewed by JS.

### Measures of treatment effect

Continuous data describing treatment effectiveness were extracted (i.e. pre- and post-treatment depression scores or change in depression score), and presented as a standardized mean difference (SMD; Hedge’s g). Values extracted were unadjusted for baseline due to unreported data. Using a random effects model, meta-analyses computed a pooled SMD with 95% confidence intervals (CI) and the *I*^2^ statistic assessing heterogeneity between studies. Data were analysed with OpenMeta[Analyst].^19^ Due to the small number of studies, sensitivity analysis was performed only on the basis of clinical heterogeneity and not statistical heterogeneity.

### Quality assessment

Included trials were assessed for the quality of methodology employed and risk of bias using the SIGN (Scottish Intercollegiate Guidelines Network) tool evaluating nine main factors: (1) appropriateness of the research question; (2) randomization; (3) concealment of allocation; (4) blinding; (5) comparability of groups at baseline; (6) comparability of follow-up (i.e. groups differ only according to treatment allocation, but no other factors that may influence outcome); (7) use of standardized measures, (8) use of intention-to-treat (ITT) analysis; and (9) differences between study sites.^20^ Overall risk of bias was rated as low, moderate or high.

## Results

### Systematic search results

**Figure 1.**
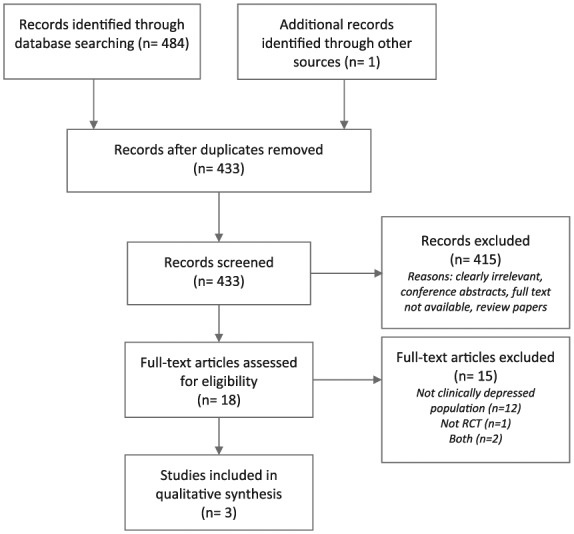
PRISMA flow chart presenting a breakdown of the search process.

Of the 485 records identified through all sources from the initial search, 433 were screened after removal of duplicates. Of these, 18 full-text articles were screened for eligibility, of which only 3 met the predefined selection criteria (see Figure 1 for PRISMA flowchart of the search process).

### Characteristics of included studies

Within the three included RCTs, a total of 229 clinically depressed patients were randomized, with an average age of 36 years and a gender distribution of 76% female. All three studies were parallel-group studies and included a placebo comparator arm. One study also included a prebiotic comparator arm.^21^ The length of intervention was 8 weeks across all studies, and in two studies the probiotic intervention was in addition to the patients’ ongoing antidepressant medication, while in one study patients were required to be psychiatric medication-free.^22^ Two of the trials were conducted in Iran and one in New Zealand. The key characteristics of the included studies are summarized in Table 1.

**Table 1.** Summary of key characteristics of the included randomized clinical trials (RCTs) (arranged by year of publication).

First author (year)	Sample	Sample size	Intervention type	Intervention length	Probiotic strains (CFU)/g and dose	Control arm(s)	Outcome measure
Akkasheh (2016)^23^	MDD patients aged 20–55	40	Add-on	8 weeks	*Lactobacillus acidophilus* (2 × 10^9^ ) *Lactobacillus casei* (2 × 10^9^ ) *Bifidobacterium bifidum* (2 × 10^9^)/g 1 capsule daily	placebo	BDI
Romijn (2017)^22^	Self-referrals with at least moderate depression score; aged >16	79	Mono-therapy	8 weeks	*Lactobacillus helveticus* *Bifidobacterium longum* (⩾2 × 10^9^)/g 1.5 g sachet daily	placebo	MADRS
Kazemi (2019)^21^	MDD patients aged 18–50	110	Add-on	8 weeks	*L. helveticus* *B. longum* (⩾2 × 10^9^)/g 5 g sachet daily	placebo prebiotic	BDI

BDI, Beck Depression Inventory; CFU, colony-forming unit; MADRS, Montgomery-Asberg Depression Scale.

### Quality assessment

Overall, the risk of bias assessment of the three RCTs was low/moderate. Two issues were identified: Akkasheh and colleagues did not report baseline depression severity for each group,^23^ which is a key variable that may influence study findings and should have been presented; Romijn and colleagues reported a significant difference in antidepressant history at baseline between groups, with 70% *versus* 46% of previous use reported in the probiotics and placebo group, respectively.^22^ However, the groups did not differ on current depression severity, or on chronicity (defined as >2 years continuous symptoms). The groups also differed in rates of irritable bowel syndrome symptoms at baseline, with the probiotic group scoring significantly higher. A breakdown of the quality assessment by study is presented in Table 2.

**Table 2.** Methodological quality and risk of bias in the included trials.

Criterion	Akkasheh et al.^23^	Romijn et al.^22^	Kazemi et al.^21^
Appropriateness and focus of study question	+	+	+
Assignment to treatment groups is randomized	+	+	+
Adequate concealment of allocation	+	+	+
Patients, clinicians and assessors are blinded	+	+	+
Similarity of groups at baseline	?	–	+
Comparability of groups during treatment period	+	+	+
Use of standardized outcome measure	+	+	+
Intent-to-treat analysis	+	+	+
Comparability between study sites	n/a	n/a	n/a
Overall assessment or risk of bias	low/moderate	low/moderate	low

### Effects of probiotic treatment on depressive symptoms

**Figure 2.**
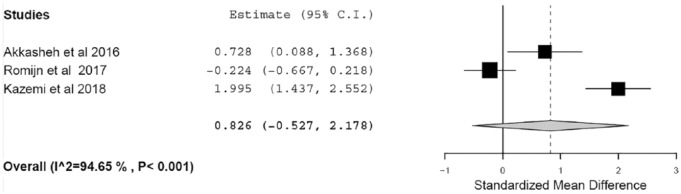
Forest plot showing standardized mean difference in depressive symptoms, comparing probiotic and placebo.

Overall, there was a nonsignificant difference in depressive symptoms between the probiotic and placebo groups postintervention (SMD = 0.826, 95% CI −0.527 to 2.178, *p* = 0.231; *I*^2^ = 94.7), with high heterogeneity between the studies observed (Figure 2).

Akkasheh and colleagues found a significant difference in Beck Depression Inventory (BDI) scores after 8 weeks of add-on treatment with probiotics, with mean reduction of BDI total score of −5.7 in the probiotics group compared with −1.5 in the placebo group (*p* = 0.001).^23^ When adjusted for baseline levels, this effect remained significant (*p* = 0.05). Kazemi and colleagues also reported a significant decrease in BDI total score in the group receiving probiotics, with a mean reduction of −9.25 compared with −3.19 in the group receiving placebo after 8 weeks of add-on treatment (*p* = 0.008).^21^ Romijn and colleagues found no significant difference between probiotic and placebo treatment groups in Montgomery-Asberg Depression Scale (MADRS) total score after 8 weeks of primary treatment with probiotics, with a mean change in MADRS total score of −8.7 in the probiotic group and −9.7 in the placebo group (adjusted for baseline; *p* = 0.62).^22^

### Sensitivity analysis

The study by Romijn and colleagues differed from the other two trials in that individuals were not taking any antidepressant medication at the time of the study.^22^ Furthermore, individuals were recruited by advertising and were not formally assessed using a diagnostic scale for depression, although they did self-report symptoms consistent with moderate MDD. Due to this clinical heterogeneity. we repeated the meta-analyses after excluding this study, which yielded a significant effect of probiotics on reducing depressive symptoms (SMD = 1.371, 95% CI 0.130–2.613, *p* = 0.03; *I*^2^ = 88.3); however, heterogeneity remained high. On the basis of reported study and sample characteristics in the remaining two RCTs, no cause for further clinical heterogeneity could be identified.

### Tolerability and adherence

Overall, probiotic supplements were well tolerated with no related serious adverse events (AEs) reported and a low drop-out rate in all studies. Akkasheh and colleagues did not report AE data.^23^ The most commonly reported AEs in the remaining two studies were gastrointestinal complaints, nausea and change in appetite. The rates of these did not differ significantly between groups in the study by Romijn and colleagues; the only AEs that were significantly different were dry mouth and sleep disruption, with higher rates reported in the placebo group.^22^ Kazemi and colleagues reported AEs occurring in 10 participants in the probiotic group and 1 in the placebo group; no comparative analyses were reported, likely due to the low AE rates observed.^21^ Adherence was high across all studies, with supplement counts showing between 90–97% of doses taken.

## Discussion

Evidence from animal models and healthy volunteer studies suggests that probiotics may be beneficial for alleviating stress, anxiety and depressive symptoms. In this systematic review we aimed to identify all RCT data on the efficacy of probiotics as treatment for depressive symptoms in clinically depressed patients. Only three RCTs met our inclusion criteria, suggesting that there is still an insufficient evidence base. The duration of intervention in all three studies was 8 weeks, and a standardized measure of depressive symptoms was employed as a primary outcome measure. Adherence to the intervention and tolerability were high in all studies. Overall, there was a nonsignificant difference in depressive symptoms between the probiotic and placebo groups postintervention. However, as discussed in the sensitivity analysis, two of the studies, which administered probiotics as add-on to ongoing antidepressant therapy in patients with MDD, reported that probiotic supplements significantly improved depression scores compared with placebo.^21,23^ In contrast, the third study, which administered probiotics as a standalone treatment in medication-free patients with self-rated moderate-to-severe depressive symptoms, reported no significant difference between the probiotic and placebo arms.^22^

In the study by Romijn and colleagues,^22^ patients self-referred to the study and completed self-rated depression measures during an online screening process. A minimum of moderate depression severity was a requirement for inclusion; however, a formal diagnostic interview or screening of medical records was not performed at baseline. Instead, current depressive state and illness history were established entirely by self-report and retrospective self-report. As the screening measures used have been well-validated in the field, this population was considered to meet criteria for clinical depression and was included in this review. As ‘depression’ is an umbrella term used for multiple disorders, the context in which participants were experiencing depressive symptoms is not clear, suggesting that this was likely to have been a heterogeneous population. This potential heterogeneity of the sample may have been a contributing factor to the lack of significance of the main effect, and also impedes the clear interpretation of the findings. Removal of the Romijn study revealed a significant effect of probiotics as an add-on to antidepressant treatment on depression. It will be important to replicate this, and to determine the underlying mechanisms explaining why add-on therapy may be effective whereas monotherapy is not. Similarly, to evaluate other potential clinical sources of variance, it would have been informative if the two add-on studies provided data on the duration, dose and level of prior response to the ongoing antidepressant treatment. Further, baseline depression severity was not reported in one of these studies,^23^ which may have contributed to the high heterogeneity observed here and also increases the risk of bias. It is not possible to say at this stage whether probiotics may be effective for all severities of depression.

The probiotic supplements used in the three RCTs, and in previously published studies, vary significantly, which reflects the lack of consensus in the field as to which specific strains/combinations of strains are most likely to produce antidepressant effects. Strains from the *Lactobacilli* and *Bifidobacteria* genera have most frequently shown beneficial effects in animal studies and nonclinical populations.^16^ The selection of bacteria in the three RCTs was also based on this evidence. Further studies in clinical populations are needed to identify the optimal content, dosing and duration of probiotic supplement treatment. Some have suggested that research needs to focus on identifying specific strains that are related to specific symptoms,^16^ although this is likely to be challenging due to the complexity of interactions within the gut. Further studies investigating potential antidepressant mechanisms of different strains of probiotic bacteria in humans are needed.

### Strengths and limitations

A key strength of this review is the clear and rigorous study selection criteria. Previous reviews have included all studies in humans, irrespective of illness status or design, which is of limited value in determining the clinical utility of probiotics for the treatment of depression. To our knowledge, this is the first study systematically evaluating evidence on the effectiveness of probiotics in clinically depressed individuals. The limitations of this review stem from the limited number of published RCTs. As described above, there was heterogeneity between the studies, which reduces the capacity to draw clinically meaningful conclusions, including: type of intervention (add-on *versus* standalone), intervention content (strain combinations and dosing), patient population (confirmed diagnosis of MDD *versus* self-report), depression severity threshold for inclusion (mild *versus* moderate), and AE reporting (one study did not report AE rates). Further, due to the different exploratory analyses performed in each trial, the evidence base regarding the mechanisms of action of probiotics in clinically depressed patients remains too limited for comparative analyses.

## Conclusion

There is a limited amount of evidence for the efficacy of probiotics in clinical depression, although the published data suggest there may be a beneficial effect of probiotics on depressive symptoms in MDD when administered as an add-on to antidepressants. Further larger studies in well-defined clinical populations are needed, both to determine the clinical utility of this novel treatment approach, and to investigate potential underlying mechanisms.
